# Gerechtigkeit und soziale Determinanten der Gesundheit im Lebenslauf

**DOI:** 10.1007/s00108-024-01771-7

**Published:** 2024-09-10

**Authors:** Franz Kolland, Rebekka Rohner

**Affiliations:** Kompetenzzentrum Gerontologie und Gesundheitsforschung, Karl Landsteiner Privatuniversität für Gesundheitswissenschaften, Dr.-Karl-Dorrek-Straße 30, 3500 Krems, Österreich

**Keywords:** Gesundheitsbezogene Gerechtigkeit, Soziale Diskriminierung, Intersektionalität, Gesundheitsversorgung/soziale Faktoren, Altersdiskriminierung, Health equity, Social discrimination, Intersectionality, Healthcare/social factors, Ageism

## Abstract

**Hintergrund:**

Der soziostrukturelle und soziokulturelle Wandel in den westlichen Gesellschaften stellt die Gesundheitseinrichtungen zunehmend vor Herausforderungen, die Gesundheit und Würde der Menschen gut zu berücksichtigen. Weitere und nachhaltige Fortschritte in der Gesundheitsversorgung sind zunehmend von soziokulturellen Bedingungen beeinflusst. Werden diese Bedingungen unzureichend berücksichtigt, sind die weiteren medizinischen Fortschritte gefährdet.

**Ziel der Arbeit:**

Das Ziel dieser Arbeit liegt darin, die Bedeutung sozialer Bedingungen der Gesundheit im Lebenslauf herauszuarbeiten und damit eines der vier ethischen Prinzipien in der Medizin, nämlich die Gerechtigkeit, genauer auszuleuchten.

**Material und Methoden:**

Bearbeitet wird die Fragestellung anhand sozialwissenschaftlicher Literatur, wobei die Literatur unter strukturtheoretischer Perspektive gesichtet wurde.

**Ergebnisse:**

Fühlen sich Menschen in Hinsicht auf Alter, Geschlecht oder Migrationshintergrund diskriminiert, dann hat das nicht nur Auswirkungen auf das Selbstwertgefühl, sondern auch auf die Gesundheit bzw. Rekonvaleszenz nach Erkrankungen. Ungünstige wirtschaftliche Lebensverhältnisse wirken sich auf das Gesundheitsverhalten negativ aus. Diskriminierungserfahrungen im Gesundheitswesen können die Zufriedenheit mit Behandlungen verringern und dazu beitragen, dass Behandlungsvorschriften nicht eingehalten werden.

**Schlussfolgerung:**

Beeinflusst werden können die angeführten soziokulturellen Effekte nicht nur über individuelle Verhaltensänderungen, sondern insbesondere auch über strukturelle bzw. institutionelle Wandlungsprozesse. Es braucht eine „Habitussensibilität“ sowohl im klinischen als auch im niedergelassenen Bereich, das heißt, die Verantwortung von Ärzt*innen im Gesundheitssystem liegt auch in der Beseitigung von Diskriminierung.

## Abstract (en)

**Background:**

Socio-structural and socio-cultural change in Western societies is increasingly challenging healthcare institutions to take good care of people’s health and dignity. Further and sustainable progress in health care is increasingly influenced by socio-cultural conditions. If these conditions are insufficiently taken into account, further medical progress is jeopardized.

**Aim of the paper:**

The aim of this paper is to elucidate the significance of social conditions of health over the life course and thus to shed more light on one of the four ethical principles in medicine, namely equity.

**Material:**

The question is addressed by a literature review, whereby the literature was reviewed from a structural theory perspective.

**Results:**

If people feel discriminated against in terms of their age, gender, or migration background, this not only has an impact on their self-esteem, but also on their health and recovery from illness. Unfavorable economic living conditions have a negative impact on health behavior. Experiences of discrimination in the healthcare system can reduce satisfaction with treatment and contribute to non-compliance with treatment instructions.

**Discussion:**

The socio-cultural effects mentioned above can be influenced not only by individual changes in behavior but in particular by structural and institutional change processes. There is a need for “habitus sensitivity” in both clinical and private practice, i.e., it is also part of the responsibility of doctors in the healthcare system to eliminate discrimination.

Diskriminierung in der Gesundheitsversorgung betrifft sowohl den Zugang zu Gesundheitsdiensten als auch die Qualität und Wirkung der Gesundheitsdienste. Dabei ist es jedoch wichtig, zwischen gesundheitlichen Ungleichheiten aufgrund unterschiedlicher Lebenslagen sowie diskriminierenden Handlungen und Regulierungen zu unterscheiden. In dieser Übersichtsarbeit wird das Grundprinzip der Gerechtigkeit in der Medizin in seiner sozialen Praxis reflektiert, Herausforderungen werden identifiziert.

## Ungleichheit und Diskriminierung

Gesundheit und Krankheit sind vom je eigenen Lebensstil einer Person beeinflusst. Jedoch ist dieser je eigene Lebensstil stark sozial überformt. Für Pierre Bourdieu [3] ist der Lebensstil Ausdruck des jeweiligen Habitus und symbolisiert das ökonomische, soziale und kulturelle Kapitel einer Person. Der Habitus stellt ein Wahrnehmungs- und Handlungsschema dar, das in der Sozialisation (teils unbewusst) angeeignet wird und zu einem schichtspezifischen Gesundheitsverhalten führt. Die Art, sich zu ernähren, wie man Sport treibt oder auch die Berufswahl sind also nicht nur individuelle Verhaltensweisen, sondern immer sozial geformt. Gesundheit und Krankheit sind also nicht nur von der individuellen Lebensführung abhängig, sondern Produkt gesellschaftlicher Verhältnisse und der ungleichen Verteilung von ökonomischen, sozialen und kulturellen Ressourcen [11]. Die ungleiche Verteilung von Ressourcen über den Lebenslauf führt zu Ungleichheiten in gesundheitlicher Hinsicht.

Gesundheit und Krankheit sind auch ein Produkt gesellschaftlicher Verhältnisse

Menschen in modernen Gesellschaften unterscheiden sich nach Alter, Geschlecht, ethnischer Zugehörigkeit, sexueller Orientierung, körperlicher oder geistiger Beeinträchtigung und noch weiteren soziokulturellen Merkmalen. Diese Vielfalt bedeutet zunächst einfach Verschiedenheit unter Gleichen. Diese Verschiedenheit ist aber oftmals auch mit Ungleichheit und sozialer Benachteiligung verbunden. Ungleichheit bedeutet dabei, dass sich aufgrund struktureller Unterschiede, etwa im Zugang zum Bildungs- und Gesundheitssystem und zum Arbeitsmarkt, eine ungleiche Verteilung von Ressourcen ergibt. Ungleich verteilte Ressourcen beeinflussen die Möglichkeiten, sich gesund zu verhalten, und tragen auch zu Benachteiligungen in der Gesundheitsversorgung bei [1]. Ein Beispiel wäre ein Arbeiter, der mehrere Jahrzehnte seines Lebens schwere körperliche Arbeit verrichten muss und sich aufgrund seines niedrigen Einkommens gewisse Medikamente oder Therapien nicht leisten kann.

Diskriminierung findet dann statt, wenn eine Person aufgrund eines oder mehrerer Merkmale mit Vorurteilen oder Verhaltensweisen konfrontiert ist, die ihr den Zugang zu oder die Nutzung von gesellschaftlichen Ressourcen erschwert oder unmöglich macht und damit Lebenschancen und -bedingungen ungleich beeinflusst. Vorurteile und Stereotype sind vereinfachende mentale Bilder, die zu Beurteilungen in Form sozialer Kategorisierung führen und nicht mehr individuelle Merkmale im Blick haben. Vorurteilsbereitschaft zeigt sich in verschiedenen Formen und Ausprägungen, beispielsweise in negativer Bewertung, Geringschätzung, Herabsetzung, Unterdrückung, Benachteiligung und Entwertung, dabei sind die Grenzen zum Teil fließend. Fühlen sich Menschen in Hinsicht auf Alter, Geschlecht oder Migrationshintergrund diskriminiert, hat das nicht nur Auswirkungen auf das Selbstwertgefühl, sondern auch auf die Gesundheit bzw. auf die Rekonvaleszenz nach Erkrankungen [12]. Aus einer soziologischen Perspektive ist dabei die Diskriminierung nicht nur ein individuelles Verhalten, sondern ein komplexes System historisch gewachsener gesellschaftlicher Strukturen und institutioneller Regelungen, das ungleiche Folgen für soziale Gruppen hat [19]. Die Abwertung als Ergebnis eines Diskriminierungsprozesses endet dabei nicht innerhalb einer Generation, sondern wird über Generationen weitergetragen und so dauerhaft verfestigt. Neben der negativen Diskriminierung gibt es auch eine „positive Diskriminierung“ (im Englischen als „affirmative action“ oder „positive action“ bezeichnet). Darunter kann die bewusste Bevorzugung von Personen verstanden werden, um Nachteile, denen sie ausgesetzt sind, anzugehen und auszugleichen. Derartige Ausgleichsmaßnahmen sind insofern umstritten, als sie die Menschen benachteiligen, die das entsprechende Merkmal nicht aufweisen.

In einer Stellungnahme erinnerten die Vereinten Nationen am 27.06.2017 daran [24], dass ein zentraler Grundsatz der Agenda 2030 für nachhaltige Entwicklung darin besteht, „sicherzustellen, dass niemand zurückgelassen wird“ und „die am weitesten Zurückgebliebenen zuerst zu erreichen“ sind. Die Vereinten Nationen erkennen damit an, dass Diskriminierung in der Gesundheitsversorgung ein großes Hindernis bei der Verwirklichung der Ziele für nachhaltige Entwicklung (Sustainable Development Goals [SDG]) darstellt. Viele Einzelpersonen und Gruppen werden aufgrund ihres Alters, ihres Geschlechts, ihrer ethnischen Zugehörigkeit, ihres Gesundheitszustands usf. diskriminiert, wobei sich die Formen der Diskriminierung häufig überschneiden oder verstärken. Diskriminierung betrifft sowohl die Nutzer*innen von Gesundheitsdiensten als auch die Beschäftigten im Gesundheitswesen. Sie stellt ein Hindernis für den Zugang zu Gesundheitsdiensten dar, beeinträchtigt die Qualität der erbrachten Gesundheitsdienste und verstärkt die Ausgrenzung von Einzelpersonen und Gruppen aus der Gesellschaft. Alle von den Vereinten Nationen angeführten Diskriminierungen verletzen die vier ethischen Grundprinzipien, die medizinisches Handeln in westlichen Ländern bestimmen:

- Respekt vor der Autonomie des Patienten
- Schadensvermeidung
- Fürsorge
- Gerechtigkeit

## Gerechtigkeit in der gesundheitlichen Versorgung

Kommen wir zur Gerechtigkeit. Die Weltgesundheitsorganisation (WHO) definiert Gerechtigkeit als die Abwesenheit unfairer, vermeidbarer und behebbarer Unterschiede zwischen verschiedenen sozialen, ökonomischen, demografischen und geografischen Gruppen [25]. Es geht dabei vor allem um die Verteilung von Ressourcen, und im Rahmen des vorliegenden Beitrags um die Verteilung der Ressourcen im Gesundheitssystem. Wie behandeln Gesundheits- und Pflegeeinrichtungen, Sozialversicherungsträger etc. kranke Menschen? In welcher Weise wird auf die Vulnerabilität älterer Menschen Rücksicht genommen? In diesem Zusammenhang kann auch das Prinzip des Nichtschadens genannt werden. Wenn wir auf dieser Ebene schon die Interaktion zwischen Institution und Individuum analysieren, dann ist ein Konzept wesentlich, nämlich das der Autonomie. Es geht um den Respekt vor der Autonomie.

Gesundheit und Wohlbefinden eines Menschen sind vom Moment der Geburt an (und schon davor) bis zum Tod durch soziale Prozesse beeinflusst. Die Reduzierung von Gesundheit und Krankheit auf biochemische, physiologische und genetische Prozesse – sei es durch die Medien, die Wissenschaft oder unser Alltagsverständnis – führt zu einer Unterschätzung der Bedeutung sozialer Faktoren [16].

In den vergangenen fünfzig Jahren wurden verschiedene Ansätze zur Erklärung gesundheitlicher Ungleichheit entwickelt [16]. Ein erster Erklärungsansatz geht von natürlicher und sozialer Selektion aus, das heißt gesundheitsbedingter sozialer Mobilität. Demnach sind Mortalität und Morbidität das Ergebnis sozialer Aufstiege der Gesunden und sozialer Abstiege der Kranken; Gesundheit beeinflusst den sozialen Status. Kritisch wird gegen diese These angeführt, dass die Erklärungskraft gering ist und dass sie auf zu wenige Personen zutrifft, um die gesundheitliche Ungleichheit insgesamt erklären zu können. Ein zweiter Erklärungsansatz sieht die materiellen Lebensbedingungen als Ursache von Ungleichheit. Schlechte Einkommens‑, Wohn- und Arbeitsbedingungen gefährden die Gesundheit. Dabei ist kein einzelner Faktor Ursache, sondern das Zusammenspiel ungünstiger Lebensbedingungen. Ein dritter Ansatz geht vom Gesundheitsverhalten aus, wonach Personen mit niedrigem sozioökonomischem Status eine „Kultur“ teilen, die gesundheitsschädigendes Verhalten fördert.

Schließlich soll noch die Versorgungsungleichheit genannt werden, das heißt Ungleichheiten im Zugang zu Versorgungsleistungen. Diese Ungleichheiten entstehen einerseits sozialräumlich, indem der Zugang zu medizinischen Leistungen räumlich ungleich verteilt ist, und sie entstehen andererseits durch die Gesundheitsdienstleister selbst, wenn sie eine zu geringe „Habitussensibilität“ aufweisen [18]. Gemeint ist damit beispielsweise, dass in der Kommunikation mit Patient*innen und Angehörigen diese Sensibilität fehlt. In eine ähnliche Richtung geht die „testimoniale Ungerechtigkeit“, indem Menschen in ihrer Glaubwürdigkeit ungerechtfertigt herabgestuft werden, und zwar aufgrund eines Vorurteils gegen ihre soziale Identität [5]. Empirisch nachweisbar ist auch, dass Menschen, die das Gefühl haben, aufgrund ihres sozialen Status eine schlechtere Behandlung erhalten zu haben, ungünstigere Gesundheitsergebnisse aufweisen. Diskriminierung im Gesundheitswesen verringert also die Zufriedenheit mit Behandlungen und trägt dazu bei, dass Behandlungsvorschriften nicht eingehalten werden [17].

## Altersdiskriminierung (Ageism)

Eine besondere soziale Determinante gesundheitlicher Ungleichheit stellt das Alter dar. Während Geschlecht, Religion oder ethnische Zugehörigkeit meist auf lange Dauer festgelegt sind, gilt dies für das Alter nicht. Die persönliche Identifikation mit dem Alter unterliegt einer permanenten Aktualisierung, und Älterwerden ist ein Prozess, den alle Menschen erleben können. Das führt dazu, dass Menschen bereits in jüngeren Jahren (meist negative) Vorstellungen vom höheren Alter internalisieren und versuchen, sich davon abzugrenzen. Internationale Studien zeigen, dass ältere Menschen im Gesundheitssystem anders behandelt werden und negative Altersbilder und diskriminierendes Verhalten seitens des medizinischen Personals und der Institutionen zu einer ungleichen Behandlung von älteren Menschen führen [2].

Altersdiskriminierung im Gesundheitswesen hängt mit negativen Stereotypen des Personals zusammen

So lassen sich altersbedingte Rationierungstendenzen in Österreich und Deutschland erkennen, das heißt ein Vorenthalt bestimmter medizinischer Leistungen aufgrund des höheren Alters [4]. In Großbritannien lässt sich eine Rationierung bei Programmen der Gesundheitsprävention und bei Gesundheitsdienstleistungen feststellen, beispielsweise werden Menschen ab 75 Jahren nicht mehr dieselben Behandlungsoptionen angeboten [6]. Gerade bei hochaltrigen Menschen (über 80 Jahre) zeigt sich, dass sie eine wesentlich höhere Wahrscheinlichkeit haben, nach einem Schlaganfall keine Rehabilitationsangebote zu erhalten [7]. Dies ist teilweise durch institutionelle Vorgaben auf Basis von Altersstereotypen bedingt. Im konkreten institutionellen Alltag wirken sich allerdings altersbezogene Vorurteile und damit Rationierungen vor allem dann aus, wenn begrenzte Ressourcen, wie etwa Zeit oder teure Behandlungsmethoden, verteilt werden müssen [23]. Teilweise ist dies allerdings auch auf Defizite im geriatrischen Fachwissen und auf eine ungenügende Berücksichtigung im Medizinstudium zurückzuführen [2].

Dementsprechend zeigen diverse Studien auf, dass Altersdiskriminierung im Gesundheitswesen mit negativen Altersstereotypen des Personals zusammenhängt. So lässt sich etwa feststellen, dass bei Menschen im höheren Alter gesundheitliche Probleme häufig dem biologischen Alterungsprozess zugeschrieben werden statt behandlungsbedürftigen Erkrankungen [14, 23]. In einer Befragung von Studierenden unterschiedlicher Fächer zeigte sich, dass vor allem Medizinstudierende eine negative Einstellung gegenüber hochaltrigen Menschen aufweisen [21]. Diese negativen Altersstereotype wirken sich dann auch auf den Umgang mit älteren Patient*innen aus: So neigen Ärzt:innen zu einem sogenannten „elderspeak“, das heißt, sie sprechen mit älteren Menschen anders, vor allem lauter, langsamer und bevormundend [20], was sich auch darin äußern kann, dass sie gar nicht mit dem älteren Menschen selbst sprechen, sondern mit den Angehörigen. Diese vorurteilsbehaftete Kommunikation führe nicht nur dazu, dass Patient*innen uninformiert bleiben, sondern beeinflusse auch deren Symptombeschreibung [2].

## Alter und Intersektionalität

Zentral ist darüber hinaus die Frage, wie verschiedene Ungleichheitsmerkmale (Geschlecht, Alter, ethnische Zugehörigkeit etc.) miteinander interagieren und Diskriminierung verstärken oder abschwächen. Es gilt also die Intersektionalität zu berücksichtigen. Frauen, Menschen mit Einschränkungen, Menschen mit Migrationshintergrund und ältere Menschen können von doppelten bzw. mehrfachen Diskriminierungen betroffen sein. So können zum Diskriminierungsmerkmal „Alter“ weitere Merkmale wie Geschlecht und sexuelle Orientierung hinzukommen (Mehrfachdiskriminierung). Diese Mehrfachdiskriminierung trifft in besonderem Maße ältere Frauen. Es findet nicht nur eine Fortsetzung der Diskriminierung von Frauen in der Lebensphase Alter statt, sondern durch die Zugehörigkeit zur Gruppe der alten Menschen potenziert sich die diskriminierende Situation.

Zur Diskriminierung im Gesundheitswesen aufgrund mehrerer Merkmale und vor allem aufgrund des sozioökonomischen Status gibt es kaum Studien, Ungleichheiten im Gesundheitszustand sind jedoch gut dokumentiert. Als zentrales Maß gesundheitlicher Ungleichheit gilt die Lebenserwartung, da sie den kumulierten Einfluss der Biologie, des Verhaltens und der Umwelt auf die Gesundheit ausdrückt. In ihr zeichnen sich somit unterschiedliche Arbeits- und Lebensbedingungen, unterschiedliches Gesundheitsverhalten und ein ungleicher Zugang zum Gesundheits- und Bildungssystem ab [15]. In Europa haben Männer eine höhere Mortalität und damit eine niedrigere Lebenserwartung als Frauen. Dies betrifft allerdings nicht alle Männer gleichermaßen, vor allem Männer mit niedrigerem Bildungsstand haben eine niedrigere Lebenserwartung, sterben also durchschnittlich früher. Gründe dafür liegen unter anderem in einem anderen Habitus, der ein anderes Gesundheitsverhalten bedingt [13].

Mehrfachdiskriminierung trifft in besonderem Maße ältere Frauen

Statistik Austria [22] stellt etwa für Österreich große Unterschiede in der ferneren Lebenserwartung (mit 65 Jahren) nach der höchsten abgeschlossenen Schulbildung fest. In Tab. 1 wird verdeutlicht, dass bei der ferneren Lebenserwartung Männer mit Pflichtschulabschluss eine um 4,9 Jahre niedrigere Lebenserwartung haben als Männer mit Hochschulabschluss. Der geschlechtsspezifische Unterschied in der Lebenserwartung ist derweil bei Menschen mit höherer Bildung geringer. So haben Frauen mit Pflichtschulabschluss eine um 4,4 Jahre höhere Lebenserwartung als Männer mit Pflichtschulabschluss, während die geschlechtsspezifische Differenz zwischen Männern und Frauen mit Hochschulabschluss auf 2,1 Jahre sinkt. Andererseits zeigt die Tabelle aber auch, dass trotz der Bildungsunterschiede das Geschlecht weiterhin eine große Rolle spielt. Denn Frauen mit Pflichtschulabschluss haben fast dieselbe Lebenserwartung (20,45 Jahre) wie Männer mit Hochschulabschluss (20,95 Jahre).

**Tab. 1:** Fernere Lebenserwartung im Alter von 65 Jahren nach höchster abgeschlossener Bildung 2021. (Quelle: Statistik Austria, demografische Indikatoren. Erstellt am 04.09.2023)

	Lebenserwartung ab 65 Jahren (in Jahren)	
	Männer	Frauen
Pflichtschule	16,10	20,45
Lehre/BMS (ohne Matura)	17,87	21,64
AHS, BHS, Kolleg (mit Matura)	19,44	22,33
Akademie, Hochschule	20,95	23,09
Insgesamt	17,92	21,18

*AHS* allgemeinbildende höhere Schule, *BHS* berufsbildende höhere Schule, *BMS* berufsbildende mittlere Schule

Neben der Mortalität lassen sich auch Unterschiede in der Morbidität feststellen, wobei vor allem hochaltrige Frauen eher eine oder mehrere chronische Erkrankungen aufweisen als hochaltrige Männer. Das bedeutet, Frauen werden zwar älter, allerdings nicht unbedingt in guter Gesundheit. Die unterschiedlichen Morbiditätsraten von Männern und Frauen können mit Geschlechtsrollenidentitäten erklärt werden, das heißt mit dem „gender bias in reporting“. Frauen berichten aufgrund ihres ganzheitlicheren Gesundheitsverständnisses Erkrankungen anders. Frauen sind expressiv orientiert. Männer definieren Gesundheit über ihre Leistungsfähigkeit und tendieren dazu, negative Befindlichkeiten zu verleugnen. Sie sind instrumentell orientiert [8]. Das bedeutet allerdings auch, dass ältere Frauen und Männer ein anderes Gesundheitsverhalten aufweisen und Frauen eher mit ihrer praktischen Ärztin bzw. ihrem praktischen Arzt ihre Gesundheit besprechen. Gründe für die geschlechts- und bildungsspezifischen Altersunterschiede in der Gesundheit können also einerseits im unterschiedlichen Gesundheitsverhalten ausgemacht werden, beispielsweise mit Blick auf Rauchen, Alkohol oder das Aufsuchen einer Ärztin/eines Arztes [10]. Andererseits bestehen in der Gesundheitsversorgung unterschiedlich starke Machtgefälle. So zeigt sich etwa, dass Personen mit niedrigerem sozioökonomischem Status tendenziell weniger Fragen an Ärztinnen und Ärzte stellen und dadurch weniger Informationen erhalten. Darüber hinaus stammen Ärztinnen und Ärzte meist selbst aus der Oberschicht und verwenden eine Sprache, die Personen mit niedrigerem Bildungsstand schwerer verstehen, oder sie setzen ein Wissen voraus, das nicht unbedingt vorhanden sein muss [9].

Gerechtigkeit im Gesundheitswesen ist also nicht nur eine Frage von individuellen Verhaltensweisen, sondern insbesondere von strukturellen bzw. institutionellen Prozessen, die Lebenschancen und -bedingungen sowie Vorurteile prägen und eine ungleiche und diskriminierende Verteilung von Ressourcen begünstigen. Es braucht also eine „Habitussensibilität“ sowohl im klinischen als auch im niedergelassenen Bereich.

## Ausblick: Die vergessene soziale Frage

Die Coronapandemie hat aus sozial-gerontologischer und machtsoziologischer Perspektive eine Reihe von Besonderheiten und Problemlagen hervorgebracht, die teilweise als außergewöhnlich klassifiziert werden können und damit kaum weitere Aufmerksamkeit auf sich ziehen, teilweise aber auch grundlegende und grundsätzliche Schwachstellen der Vergesellschaftung des Alters sichtbar gemacht haben. Diese Schwachstellen sind über weite Strecken nicht einmal neu, sondern waren strukturell und latent vorhanden. Sie wurden deshalb nicht schmerzhaft empfunden, weil sie schon als auf dem Weg der Überwindung eingestuft wurden. Von welchen Schwachstellen ist dabei die Rede?

Da geht es erstens um die Problematik stereotyper Altersbilder. Als besonders ärgerlich ist die Beobachtung einzuordnen, dass Alter homogenisiert wird, dass von den Alten die Rede ist, die gleichsam alle ein Risiko darstellen. In dieser Hinsicht war der gesellschaftliche Diskurs vor der Pandemie schon ein erhebliches Stück differenzierter.

Zweitens sind über die Zeit der Coronapandemie hinweg immer deutlicher Lücken und Schwächen in der Pflege- und Gesundheitsversorgung sichtbar geworden. Geradezu besorgniserregend zeigt sich der Personalmangel in der Pflege. Dieser ist auch nicht neu, aber hat durch den Krisenmodus noch einen zusätzlichen Schub erhalten.

Die soziale Frage ist – drittens – nicht nur eine Frage von sozialen Ungleichheiten oder Zugängen zu Macht und Einfluss. Die soziale Frage ist auch deshalb relevant, weil sie ganz grundsätzlich gar nicht oder unzureichend thematisiert wird. Zeigen lässt sich das an einem Megabegriff, der in der Pandemie in den Vordergrund gerückt ist, nämlich dem der Vulnerabilität. Über lange Zeit waren es fast ausschließlich gesundheitliche Überlegungen, die mit der Vulnerabilität alter Menschen verknüpft wurden. Soziale Fragen wurden ausgeblendet. Gesundheitliche Folgen von Einsamkeit und Rückzug wurden erst sehr viel später thematisiert. Nach dieser Zeit kam es zu einem Paradigmenwechsel und wirtschaftliche Überlegungen verdrängten alle anderen Fragen. Auf vulnerable alte Menschen wurde nur mehr sehr wenig Rücksicht genommen, um wirtschaftliche Interessen nicht zu gefährden. Es wurde nach Aufhebung der meisten Maßnahmen viel zu wenig in Hinsicht auf das gesellschaftliche Miteinander unternommen. Die verbliebenen Regeln haben ihre normative Kraft und Zustimmung verloren.

In der Langzeitpflege sollte auf Begriffe wie „Patient*in“ oder „Versorgung“ verzichtet werden

Was wir für die Zukunft unbedingt brauchen, ist eine sorgende Gesellschaft, die das „Sich-Kümmern“ nicht abschiebt, sondern als gemeinschaftliches, soziales Tun versteht. Was ist dabei das Ziel? Das Ziel ist Lebensqualität für alle Menschen unabhängig von ihrer sozialen Herkunft und Position im Lebenslauf. Es geht um objektive und subjektive Gesundheit im Alter. Mag es auch merkwürdig klingen, von Gesundheit im Alter zu sprechen, wenn da und dort körperliche Einschränkungen gegeben sind, so geht es gerade darum, die Gesundheit und eine positive Lebenseinstellung nicht aus dem Auge zu verlieren. In dieser Hinsicht spielen die Kompetenzen und Fähigkeiten aller Beteiligten eine wesentliche Rolle. Es geht nicht nur um die Kompetenz jener, die pflegen, sondern auch um die Kompetenzen jener, die gepflegt werden.

Bei Menschen in der Langzeitpflege sollten Begriffe wie „Patient*in“ oder „Versorgung“ nicht mehr verwendet werden. Sie deuten noch auf eine reparaturmedizinische Perspektive hin und nicht auf eine neue sozial verortete Sorgekultur. Der sorgende Umgang mit Menschen mit Pflegebedürftigkeit schließt Selbstsorge, Fürsorge, Vorsorge und Nachsorge ein, die im Kontext einer sorgenden Gemeinschaft oder auch „caring community“ stattfinden. Eine Pandemie ist nicht nur ein Gegenstand für Expert*innen, sie ist in ein soziales Geschehen eingebettet und verlangt deshalb auch eine Herangehensweise, die über Medizin und Wirtschaft hinausgeht. Gemeint ist damit eine Fokussierung auf die Ganzheitlichkeit des Menschen, auf den Menschen als eine ganzheitliche Persönlichkeit.

## Fazit für die Praxis

- Die empirische Forschung belegt, dass Diskriminierung „naturalisiert“ wird und dass Vorurteile eines natürlichen Prozesses gerade bei älteren Menschen zu ungleicher Behandlung führen. Hier könnte mehr geriatrisches Fachwissen in der Ausbildung zu weniger Diskriminierung beitragen.
- Der Leitspruch „Der Mensch im Mittelpunkt“ ist dann zu hinterfragen, wenn er einer reduktionistischen Handlungsperspektive folgt. Menschen werden oftmals nur über ihre Rolle (Patient, Arzt usw.) oder Erkrankung definiert. Der Leitspruch sollte von einer „Habitussensibilität“ – und dazu gehört auch eine testimoniale Gerechtigkeit – begleitet werden. Das heißt, Vorurteilen in der täglichen Praxis kann durch eine Sensibilisierung der Kommunikation für herrschende Machtdynamiken und soziale Aspekte der Gesundheit begegnet werden.
- Des Weiteren begünstigen institutionelle Rahmenbedingungen diskriminierendes Verhalten und Rationierungen, weshalb etwa für multimorbide, ältere Menschen verstärkt die notwendigen Strukturen ermöglicht werden müssen (Stichwort: Akutgeriatrie).
